# 3D replicon distributions arise from stochastic initiation and domino-like DNA replication progression

**DOI:** 10.1038/ncomms11207

**Published:** 2016-04-07

**Authors:** D. Löb, N. Lengert, V. O. Chagin, M. Reinhart, C. S. Casas-Delucchi, M. C. Cardoso, B. Drossel

**Affiliations:** 1 Department of Physics, Institute for Condensed Matter Physics, Technische Universität Darmstadt, 64289 Darmstadt, Germany; 2 Laboratory of Chromosome Stability, Institute of Cytology, St Petersburg 194064, Russia; 3 Department of Biology, Technische Universität Darmstadt, 64287 Darmstadt, Germany

## Abstract

DNA replication dynamics in cells from higher eukaryotes follows very complex but highly efficient mechanisms. However, the principles behind initiation of potential replication origins and emergence of typical patterns of nuclear replication sites remain unclear. Here, we propose a comprehensive model of DNA replication in human cells that is based on stochastic, proximity-induced replication initiation. Critical model features are: spontaneous stochastic firing of individual origins in euchromatin and facultative heterochromatin, inhibition of firing at distances below the size of chromatin loops and a domino-like effect by which replication forks induce firing of nearby origins. The model reproduces the empirical temporal and chromatin-related properties of DNA replication in human cells. We advance the one-dimensional DNA replication model to a spatial model by taking into account chromatin folding in the nucleus, and we are able to reproduce the spatial and temporal characteristics of the replication foci distribution throughout S-phase.

## Abstract (web summary)

DNA replication in higher eukaryotes is a complex process occurring in a complex genome environment. Here the authors present a model of DNA replication that incorporates random loop chromatin folding and domino-like fork progression reproducing the spatial and temporal characteristics of S-phase.

## Graphical abstract

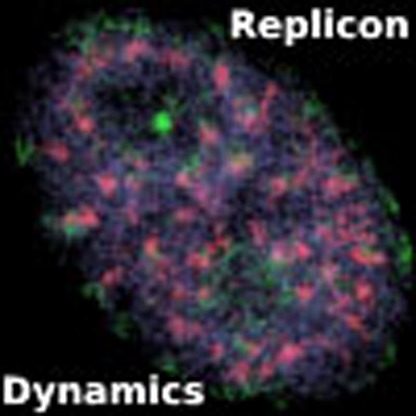

When the genome of eukaryotic cells is duplicated during the S-phase of the cell cycle, it is essential that the entire karyotype is reliably and precisely reproduced. Importantly, this process must be able to cope with variations in S-phase duration^1^, potential chromosomal abnormalities and ploidy variations. Before the actual replication start, pre-replicative complexes are assembled on the DNA, licensing the origins of replication initiation^2^. These origins are activated by specific proteins, which initiate DNA duplication by interacting with the DNA polymerase complex^3^. The sites of DNA synthesis are called replication forks, which normally emerge in bidirectional pairs from each activated origin and travel in opposite directions. The DNA segment duplicated by such a pair of replication forks is termed as ‘replicon'^3^,^4^. The amount of time needed to duplicate a DNA molecule depends solely on the speed of replication fork movement and the sum and distribution of activated origins.

Metazoan genomes feature a higher order organizational structure, which is not present in the well-characterized yeast model organisms^5^,^6^,^7^. Contrary to yeast, the positions of replication origins in metazoan DNA do not appear to be determined by DNA sequence^8^,^9^. Positions and activation times of individual origins can be related to various chromatin features^3^,^10^,^11^,^12^,^13^,^14^, and molecular analyses have shown that positions of active origins, inter-origin distances and the speed of replication fork movement can vary even within individual cells^15^,^16^. Biological analyses of replication progression throughout S-phase in mammalian cells led to a domino-like next-in-line model^17^ where replication is triggered by replication of adjacent regions. Guilbaud *et al*.^18^ described chromosomal regions in HeLa cells with sequentially activated origins that are neither clearly early nor clearly late replicating. The existence of a long-range control of otherwise stochastic or induced firing of origins in the presence of replication forks was subsequently suggested. Genome-scale mapping of DNA replication origins demonstrated general plasticity of active origin positions, which was interpreted as replicon size flexibility within a predetermined replicon cluster^19^. Accordingly, the replication programme in metazoans demonstrates a high level of plasticity, thus ensuring complete genome duplication in the face of developmental and environmental changes^1^. Models of genome duplication in metazoans, therefore, need to include stochastic mechanisms to account for origins initiated at non-predetermined sites^20^ and a flexible spatio-temporal structure of S-phase^13^,^21^. Recently, a quantitative model of human genome replication was presented by Shaw *et al*.^22^. By introducing clusters of origins which are fired together spontaneously or by activation from a neighbouring cluster, and by implementing the observed temporal variation of fork speed^23^, the authors reproduce S-phase dynamics and replication progression on a cluster scale. However, the formation of clusters is likely to emerge from more elementary processes. The interplay of deterministic and stochastic influences in these processes, which is yet unclear^24^,^25^, needs to be motivated by more detailed experimental data. Besides, an adequate model of genome duplication in eukaryotes must reproduce not only the temporal dynamics, but also the spatial characteristics of DNA replication *in vivo*. Here, we use domino-like DNA replication progression and random loop folding of chromatin to present a minimal model of DNA replication in higher eukaryotes that is able to reproduce spatial dynamics of the replication foci (RFi) throughout S-phase without need for replicon clustering at common synthetic centres as shown in Chagin *et al*.^26^

## Results

### Correlated and limited firing of origins

Potential replication origins are distributed randomly on the DNA at distances down to a few kbp (refs 2, 7, 18, 27) and are capable of firing spontaneously. Thus, in our model the location of potential origins along the DNA is determined randomly. The probability for spontaneous firing events is assumed to be higher in euchromatic regions than the probability of firing potential origins in facultative and constitutive heterochromatic regions. Further firing events are ‘induced' events in the proximity of active replication forks^17^.

**Figure 1:**
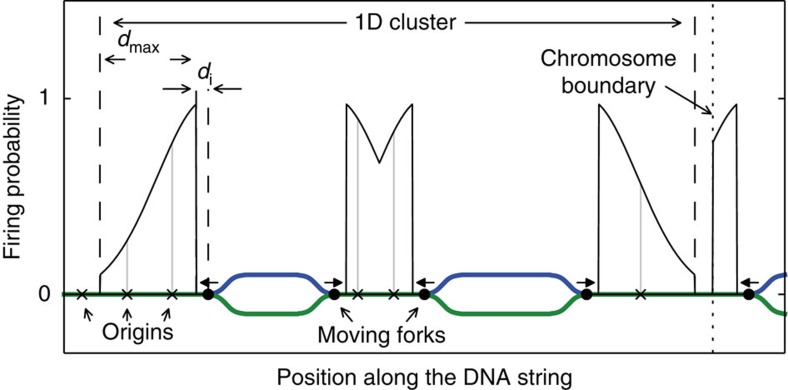
Induced firing probability. The firing probability of origins that are close to forks follows a Gaussian probability density, indicated as shaded areas next to the forks. Firing at positions closer than *d*_i_=55 kbp to a fork is inhibited and the probability density is cutoff at values below 0.1. The relative probabilities of individual origins are indicated by dark grey bars. All four forks to the left of the chromosome boundary belong to a single 1D fork cluster (assuming that neighbouring forks are <1 Mbp apart). The chromosome boundary near the right edge of the image isolates chromatin belonging to different chromosomes and thus cuts off the induced firing range of the rightmost fork.

**Figure 2:**
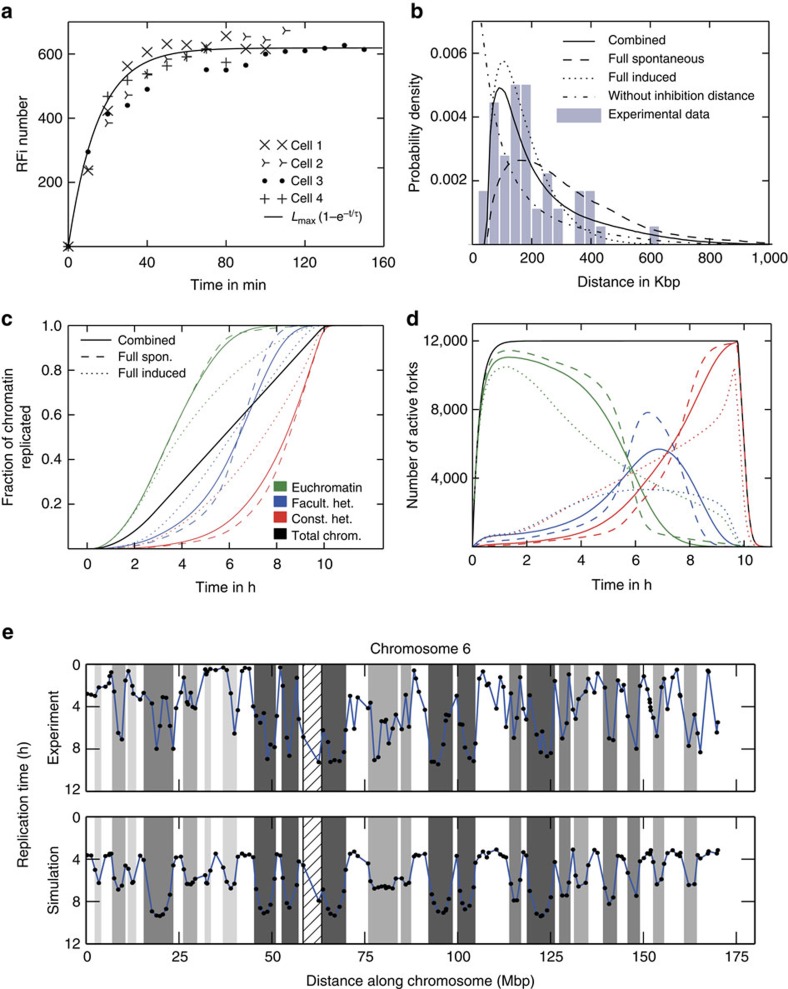
Several simulated replication characteristics compared with experimental data. (**a**) Confocal RFi measurements were used to model the initial increase of the limiting factor with a mono-exponential fit *L*(*t*)=*L*_*max*_(1−*e*^−*t*/*τ*^) with timescale *τ*=15 min. (**b**) Distribution of distances between adjacent fired origins from DNA combing data for HeLa Kyoto cells. The distribution has a peak below 200 kbp and a heavy tail up to 600 kbp. The corresponding distribution, averaged over 100 simulations, displays similar features. (**c**) Fraction of replicated chromatin as a function of time. Colours are used to distinguish between the chromatin type specific and total replication. Dotted lines show the simulation results, when only induced firing events are allowed. Dashed lines display the other extreme case, where solely spontaneous firing was used. The combined model includes both firing events and the results are shown with solid lines. (**d**) Time-dependent number of forks in each chromatin type. (**e**) Comparison of our model with replication timing data for chromosome 6 from the ENCODE project^44^ (cell type GM12878). Sampling positions are identical to the positions in the experimental data. For individual simulations, the euchromatic peaks start at time zero, but because of the specific sampling positions and averaging over 100 simulations, the displayed peaks are less extreme. The Pearson's correlation coefficient between the theoretical and experimental data shown here is 0.60. The Background indicates the Giemsa staining, where white regions are interpreted as euchromatin and shaded regions as facultative or constitutive heterochromatin. The centromere is indicated as a striped pattern. Analogous figures for other human chromosomes can be found in the Supplementary Figs 4–6.

Due to the induced firing process, the probability for very short distances between firing origins would be much higher than experimentally observed (Fig. 2b). Thus, we introduced a distance around active forks, where firing of potential origins is inhibited (the inhibition distance—*d*_i_). A range of the *d*_i_ values from 7 to 120 kbp was selected based on the reported correlation of distances between preferentially activated origins^27^,^28^,^29^,^30^,^31^ and average sizes of the chromatin loops in different functional chromatin organization models^14^,^32^. To find the most probable value for *d*_i_ we compared the experimental distribution of inter-origin distances (Fig. 2b) with the distribution obtained from simulations varying the *d*_i_ value (5 kbp steps) by calculating the *χ*^2^ value as well as the Kullback–Leibler divergence. Both measures have a broad minimum for *d*_i_ values between 35 and 55 kb indicating the most probable range. In the simulations presented here, a value of 55 kb was used, because smaller values lead to an increasing total number of origins fired. Figure 1 shows a schematic of the induced firing process in the model. The range of induced firing is determined by the parameter *σ*, the s.d. of the Gaussian curve, which is used to set the induced firing probabilities of nearby potential origins. Induced firing probabilities below 0.1 are set to zero to avoid the infinite range of the Gaussian curve. Increasing the value of *σ* broadens the simulated distribution of inter-origin distances shifting the mean towards higher distances and decreasing *σ* enhances the peak of the distribution below 200 kb. In the range from 100 to 280 kb for the parameter *σ* there are only minor changes to the distribution of inter-origin distances, therefore it can not be determined more precisely from the given data.

The shape of the DNA flow cytometry histogram and equal replicon numbers throughout S-phase^26^ suggest a rate of global DNA duplication approximately constant through most of S-phase. This is modelled by introducing a ‘limiting factor', representing a necessary component of each active replication fork, that limits the total number of active replication forks in the nucleus to the number of the limiting factor molecules. The concept of a limiting number of available forks was also used in models of metazoan DNA replication to obtain realistic origin activation profiles and synthesis rates^33^,^34^,^35^. We assume that the limiting factor moves nearly instantaneously through the nucleus^36^,^37^, starts to become available once the cell enters S-phase, and that its number increases during the first hour until it reaches a maximum level that is maintained until the end of S-phase (Methods section and Fig. 2a). Our experimental data suggest that the number of simultaneously active replicons is between 4,000 and 6,000 (ref. 26), which is of a similar order of magnitude as previous DNA replication models suggested^33^,^34^. Hence, the maximum number of active replication forks is set to *L*_*max*_=12,000. The total genome replication time of 10.3 h obtained in the computer simulation using this limiting factor concurs with the empirically found S-phase duration of 9.5 h±0.8 (s.d.)^26^.

It is estimated that the total number of active origins involved in the replication of an entire mammalian genome lies in between 30,000 and 50,000 (refs 19, 26, 38), which includes the simulated value ranging from 43,800 to 44,500 (simulation parameters listed in Table 1). In simulations with smaller inhibition distances the total number of fired origins increases up to a value of 74,000 at *d*_i_=0. Thus our model predicts, that not more than one origin is activated per chromatin loop with a passive replication of other potential origins in the loop^28^,^30^, which reproduces the known correlation between the replicon and chromatin loop sizes.

### Slower replication in early S-phase

**Figure 3:**
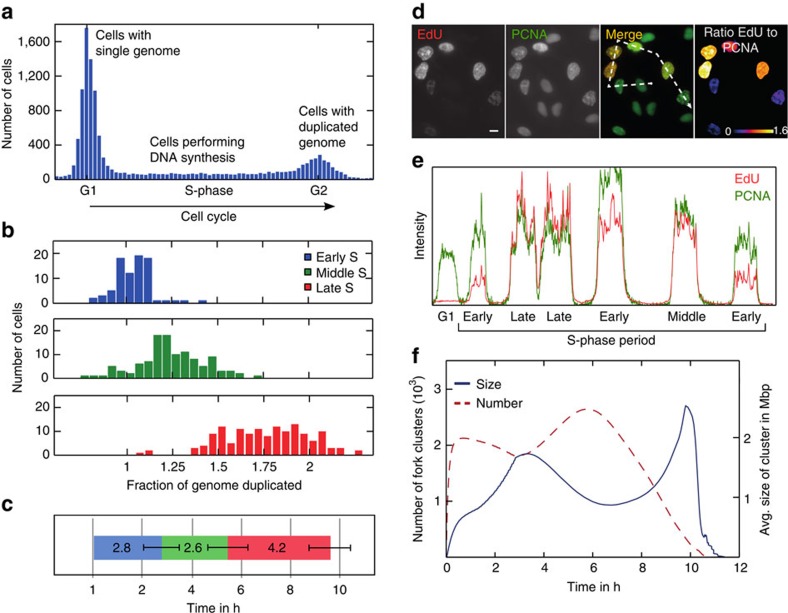
Replication subphase analysis and simulated fork clusters. (**a**) DNA content frequency throughout the cell cycle. Cells are binned by DNA content (DAPI signal), with the abscissa showing the DNA content of the bins in arbitrary units. The distribution remains at an approximately constant value throughout S-phase, that is, between the G1 and G2 peaks, meaning that the overall rate of replication is constant. (**b**) Frequency of specific DNA content intervals in an ensemble of 840 HeLa Kyoto cells from five separate slide areas dependent on their cell cycle position. Through inspection of the PCNA signal, the cells were sorted into early, middle and late S-phase. It is notable that the number of early S-phase cells drops off steeply at 15% of the DNA replicated. (**c**): The observed subphase durations, where the error bars indicate the s.d. Subphase durations were obtained using live cell microscopy as described in the accompanying manuscript Chagin *et al*.^26^ modified from Reinhart *et al*.^69^ (**d**) HeLa Kyoto cells stably expressing mCherry-PCNA were labelled with modified nucleotides (20 μm EdU) for 15 min before fixation. Wide-field images of cells going through different S-phase stages show that, while the overall level of PCNA is rather constant, the total amount of incorporated nucleotides is clearly lower in cells going through early S-phase, indicating a lower synthesis rate. From left to right: single channel images, overlay of EdU (red) and PCNA (green) signals, representation of the ratio of EdU to PCNA signal intensity. LUT as indicated. Scale bar, 10 μm. (**e**) Line profile from the overlaid EdU (red) and PCNA (green) image over six cells going through different S-phase sub-stages as indicated. (**f**) Number and size of replication clusters over time. EdU, 5-Ethynyl-2′-deoxyuridine; LUT, Lookup Tables; MCM, Minichromosome maintenance protein complex; UCSC, University of California, Santa Cruz.

We directly measured the amount of genomic DNA synthesized in each S-phase sub-period corresponding to the three major S-phase patterns (for details see Fig. 3, Methods section and Supplementary Fig. 1). Nuclei with early S-phase patterns contained up to 15% more DNA as compared with G1 population, with the cells displaying mid S-phase containing up to 50% more DNA, whereas the cells with late S-phase patterns ranged from 50% more DNA to 100%, that is, duplicated genomic DNA content (Fig. 3b). Comparing the amount of genomic DNA synthesized by RFi in a particular S-phase sub-periods with their absolute durations revealed twofold reduced global genome duplication rate in early S-phase, which lasts for 27% of S-phase (Fig. 3a–c). This observation was further supported by twofold reduced nucleotide incorporation in early S-phase pattern (Fig. 3d,e), indicating that a reduced fork speed causes the observed reduction in total DNA synthesis rate. The reduced DNA synthesis rate could be a consequence of nucleotide scarcity at the beginning of S-phase, or of the interplay between replication and transcription leading to a slower replication fork speed^39^,^40^, both of which will have the same macroscopic manifestations. We modelled slower replication in early S-phase using a linear increase in fork speed during the first 2.8 h. After the initial increase the fork speed stays constant at a value of *ν*=28 bp s^−1^ for the rest of S-phase as observed experimentally (Fig. 3a). The value was directly measured by Chagin *et al*.^26^ and is consistent with the duration of S-phase. The initial increase in the simulations was adjusted to reproduce the measured fraction of 15% (1.6 Gbp) of replicated DNA during the first 2.8 h with reduced fork speed (Fig. 3b,c). The remaining 8.8 Gbp are replicated at the full speed in ∼7.5 h, resulting in a total S-phase duration time of 10.3 h, similar to measured S-phase duration^26^ (Fig. 3a–c).

Therefore, the combination of a limiting factor and an initial fork speed increase during the first third of S-phase followed by an approximately constant rate for the rest of S-phase^10^, leads to a cell cycle profile consistent with our experimental data.

### Occurrence of a distinct mid sub S-phase

To test whether both spontaneous and induced firing are required, we varied the parameters relating to the two types of firing. The results of the two extreme cases with only spontaneous or only induced firing events are shown as dashed and dotted lines, respectively, in Fig. 2c,d.

If firing of origins is solely simulated by spontaneous events, the average replication time of the chromatin types depends highly on the spontaneous firing probabilities *p*_eu_, *p*_fac_ and *p*_con_ for euchromatin, facultative and constitutive heterochromatin, respectively. As seen in Fig. 2d a clear distinction between the subphases, in which a majority of replication forks can be found in one chromatin type, can be reproduced with probabilities satisfying *p*_eu_≫*p*_fac_≫*p*_const_ (see Table 1 for values used). However, a full spontaneous model leads to longer average inter-origin distances than experimentally observed^26^ (Fig. 2b). Increasing the differences beween the spontaneous firing probabilities does not lead to a noticeable lower average since intra chromatin zone firing still allows for very large distances shifting the distribution to larger values. To test whether the difference between the distributions is sufficient to reject the purely spontaneous model, we performed a bootstrap significance test, where the average over a subset of 50 simulated distances between fired origins was calculated. The subset was chosen randomly 10,000 times and the *P* value for the null hypothesis (no rejection) was determined from the smaller one of the fractions of simulated averages below/above the experimental average of 188 kbp. For the purely spontaneous model, after 10,000 repetitions not a single average distance greater than the experimental average was observed leading to a clear rejection with a *P* value <10^−4^.

In the case where only induced firing was allowed, the necessary initial firing was simulated by firing one origin in every euchromatic zone at the time *t*=0. This leads to good agreement with experimental data (Fig. 2b) regarding distribution of distances between fired origins (*P* value 0.20), but this scenario does not produce a visible peak of active forks in facultative heterochromatin during mid S-phase (Fig. 2d).

Thus, only a model which uses a combination of both spontaneous and induced firing reproduces correctly the distribution of distances between fired origins and a distinct middle S-phase during which mainly facultative heterochromatin is replicated. The fraction of total spontaneous firing events in the combined model is 20%, of which 92% occur in euchromatic zones. The average over a subset of 50 simulated distances between fired origins, which can be directly compared with the experimental mean value of 188 kbp (Fig. 2b), ranges from 140 to 300 kbp, depending on the predominant chromatin type. The *P* value of 0.12 obtained from the same bootstrap significance test described above is too high to reject the null hypothesis.

### Development of 1D clusters

Induced firing events in the vicinity of active forks lead to clusters of active forks on the one-dimensional (1D) DNA string, which expand outwards. As the 1D cluster increases in size, the probability that the next firing event will occur in it or close to it increases also. Clustered replication is maintained in our model through individual firing and annihilation events. We consider two adjacent forks to belong to the same cluster if their distance is <1 Mbp, which is consistent with the distance over which induced firing can occur in our model and the characteristic size of chromatin domains^41^,^42^. Clusters can therefore split into two parts that move in opposing directions when large stretches of DNA within them have been replicated. Figure 3f show the number and size of clusters during S-phase using the combined model. Spontaneous firing is dominant in euchromatin and the number of clusters increases rapidly during the initial phase (Fig. 3f) due to an increasing limiting factor and the random placement of origins over long distances. As long as the fork speed increases (until 2.8 h) the probability for neighbouring clusters to merge rises leading to a reduced cluster number. During mid S-phase clusters start splitting into two and thus the number increases again.

Measurements of the cluster size during early S-phase report a typical size of 1 Mbp (ref. 43). In our simulations the average size of replication clusters is comparable (Fig. 3f), but varies during S-phase. There is a transient increase in cluster size between 2 and 5 h caused by the spreading of early replication clusters followed by a decrease due to splitting of clusters. Since the combined model includes a very low spontaneous firing probability for origins in heterochromatic zones, most heterochromatin has to be replicated by fronts of clusters entering from adjacent zones. As the replication of smaller zones is completed the size of the remaining clusters increases because the total fork number stays constant.

### Replication front progression

We performed an evaluation of replication timing at the chromosome scale in our simulations and compared it with the microarray data from Woodfine *et al*.^10^ as well as data from the ENCODE project^44^. To mimic those experiments, we extracted the replication times of DNA corresponding to experimental sampling positions. Figure 2e shows the resemblance of our results to the experimental data. Both theoretical and experimental replication timing profiles exhibit distinctive peaks due to early replication in the euchromatic zones, including the smallest euchromatin zones. The presence of these peaks in the experiment indicates that indeed there are early firing events in all euchromatic zones, corresponding to a high spontaneous firing probability.

While our curves are averaged over 100 simulations and are therefore smooth compared with the averages of four experimental measurements, the simulated patterns still correspond to the empirical data. The centres of euchromatic regions are on average replicated first and the centres of heterochromatic regions are replicated last with distinctive transition zones in between. The model further shows groups of contiguous chromatin zones collectively replicating earlier or later than others similar to the experimental data (Fig. 2e, between 25 and 45 Mbp). On the scale of chromatin zones the replication timing profile and the number of 1D replication clusters was not sensitive to small variations in parameters, and the distribution of chromatin zone sizes as long as both firing types were enabled the majority of zone sizes was between 1 and 6 Mbp (Supplementary Fig. 2).

The correlations between simulation and experiment are comparable to the lowest correlations measured between different experiments (Supplementary Note 1 and Supplementary Table 1). We suggest that these differences are due to the given resolution of chromatin zones and the difference in specific facultative heterochromatin composition and karyotype as all experiments are based on cancer cell lines with non-diploid genomes. A more accurate agreement with empirical replication timing patterns can be achieved when local firing probabilities along the entire DNA are based on the concentration of DNase hypersensitive sites^34^, and not merely on the chromatin type.

Our model also explains how induced firing at the cluster front leads to a much higher front progression speed compared with the measured speed of single forks^26^. We obtain a cluster front speed of 100 bp s^−1^ (see Methods section for calculation), which matches the slopes of replication timing measurements reported in the literature^10^,^45^.

### Emergence of 3D replication patterns from replicon dynamics

**Figure 4:**
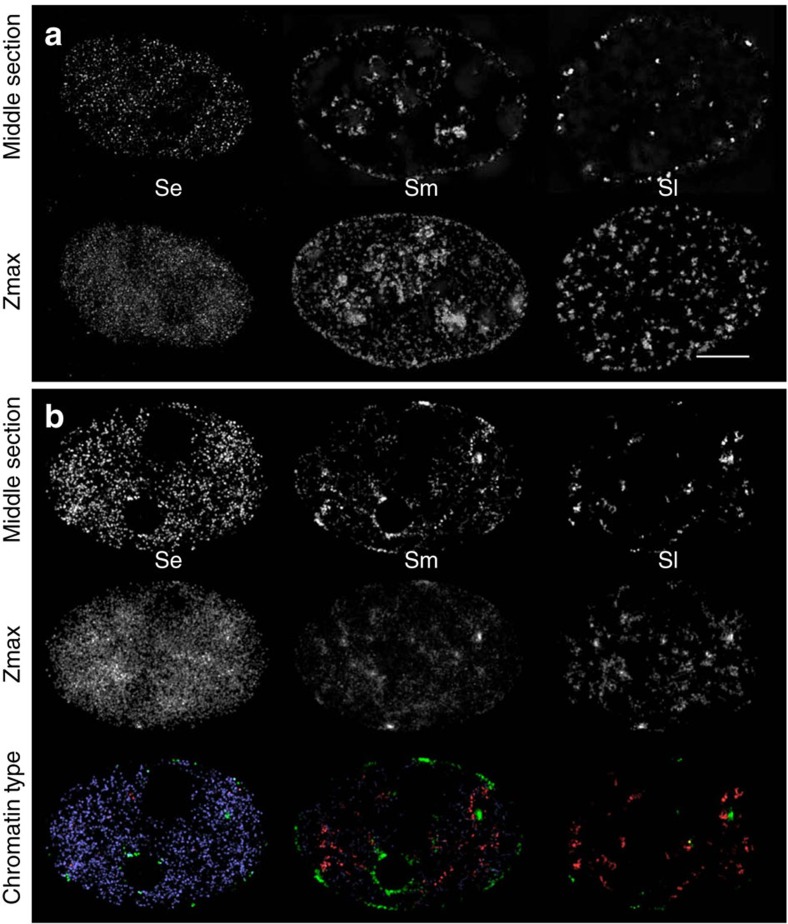
Comparison between the microscopy pattern during replication in experiment and model. (**a**) Experimental maximum intensity z-projections and middle section images of green fluorescent protein (GFP)-tagged PCNA in HeLa cells during replication (as described by Chagin *et al*.^26^ scale bar, 5 μm). (**b**) The corresponding patterns of the replication model results from a 3D DNA conformation calculated using the random loop model. The fork positions in the simulations were accumulated over 15 min similar to the experimental staining time. A Gaussian blur was applied to imitate the limited experimental voxel sizes of 40 × 40 × 125 nm. In the last row the simulated fork positions are marked depending on the chromatin type (blue, euchromatin; green, facultative heterochromatin; red, constitutive heterochromatin). Images for different parameters and chromatin distributions can be created online at http://sim.bio.tu-darmstadt.de. See also Supplementary Movies 1, 2, 3 for a visualization of the fork movement within the nucleus.

It is known from fluorescence microscopy in fixed^21^,^26^,^46^,^47^,^48^ and living cells^49^,^50^ that each of the sub-periods of S-phase is characterized by distinct patterns in the three-dimensional (3D) nuclear arrangement as well as by different clustering of RFi. To compare the dynamics of the 1D replication clusters in our model with the experimentally observed 3D characteristics of RFi, we generated in silico microscopy images of our model results (Fig. 4). To this purpose, we created a Monte Carlo simulation based on the random loop model for long polymers by Bohn *et al*.^51^, which has already been successfully used to describe folding of chromatin in human cells^52^.

Under the assumption of different chromatin compaction for particular chromatin types^11^,^53^,^54^, a combination of higher spring constants in heterochromatin with truly random linking results in chromosomes with dense heterochromatic regions and a wider nuclear region containing primarily euchromatin. We extended the random loop model to include the experimentally observed accumulation of facultative heterochromatin at the nuclear and nucleolar periphery by simulating a cell with two nucleoli, inaccessible for the polymer chain. A pseudo gravitational potential was used to attract facultative heterochromatin to the nuclear and nucleolar periphery and the same force with reverse sign also causes the distribution of constitutive heterochromatin in the bulk of the nucleus. Additionally, a small repulsive force was introduced into the model to minimize the overlap of chromosome territories.

Microscopy images of early S-phase show a large number of small and evenly distributed RFi in the entire nuclear volume except nucleoli. During early S-phase in our simulations most forks as well as 1D fork clusters are within euchromatin. The decreased compaction of euchromatin together with the considerable size of 1D clusters gives the fork distribution a seemingly random pattern resembling early S-phase microscopy images as described above. The arrangement of foci at the nuclear and nucleolar periphery observed during middle S-phase in the experiment is also reproduced by our model. In this subphase the simulation places most of the active replication forks in facultative heterochromatic zones followed by a gradually increasing number of active forks in constitutive heterochromatin. Hence, the mid S-phase pattern is generated by a superposition of both the facultative and the constitutive heterochromatic patterns in the 3D DNA conformation. Replication of facultative heterochromatin, especially inactive X chromosome replication, occurred during mid S-phase in a shorter time interval compared with the other chromosomes in agreement with experimental findings^11^. In our replication model, forks during late S-phase are located primarily in heterochromatin, which in the random loop model is constrained to a small volume for each chromosome. When the 1D replication clusters are therefore concentrated in the heterochromatin zones of a chromosome, 3D clusters characteristic for the empirical late S-phase patterns are formed. The high density of replication forks within 1D clusters during late S-phase amplifies this effect further. While we observe a steady increase in the size of replication clusters, these results demonstrate that 3D RFi distribution is primarily determined by their localization in particular chromatin types (Fig. 4b).

The simulations can be performed online at http://sim.bio.tu-darmstadt.de with a custom set of parameters and various chromatin type distributions. Graphs for visualization of the results as well as 3D in silico microscopy images are created online. Also, videos of the fork movement inside the whole nucleus (similar to Supplementary Movies 1, 2, 3) and of the fork progression on a single chromosome are available online.

## Discussion

We have demonstrated that a stochastic model of domino-like DNA replication progression reproduces the spatio-temporal characteristics of replication dynamics in human cells. The model involves a minimalistic set of parameters, derived from experimental data in HeLa cells, and independently includes the rules for DNA replication initiation, the distribution of chromatin zone sizes^55^ (Supplementary Fig. 2) and a random loop higher order chromatin organization^51^,^52^. Our model is minimal also in the sense that it reproduces S-phase dynamics in four-dimension on the basis of initiation rules for individual replicons and spatial chromatin arrangement independent from any common synthetic centres such as replication factories as shown in Chagin *et al*.^26^

A central mechanism of our model is the domino-like effect of firing of origins occurring in the proximity of active replication forks^17^. The inhibition distances of 55 kb was selected within the range of known sizes of chromatin loops involved in DNA replication^14^,^28^,^30^,^31^ and fits already described evidence regarding preselection of origins to be activated. In Jun *et al*.^14^, the origin spacing and initiation rate has been linked to chromatin loop formation probability determined by persistence length of the chromatin. The process of DNA replication in Xenopus early embryo was modelled within the paradigm of chromatin loops fluctuating around replication factories, where the probability of particular origin initiation depended on the distance to the two left and right approaching forks^14^. We do not use the concept of replication factories in our simulations, but rely on chromatin looping in determining the inhibition distance that corresponds to ‘origin exclusion zone' discussed earlier^56^. Biological effects behind inhibition of firing ahead of active replication forks can include topological constraints preventing DNA unwinding at proximal origins and/or mechanisms preventing replication machinery assembly at the sites, which are not at the bases of chromatin loops. In the first scenario the size of these replication-related loops will be mainly determined by stiffness of the chromatin fiber around the active replication forks, while the second scenario implies that looping pattern of chromatin can be predetermined in G1. Thus, our model incorporates assembly of replication initiation factors at chromatin loop bases, but spatial and temporal dynamics of genome duplication is reproduced without the concept of multiloop aggregates assembled around replication factories and corresponding clusters of synchronously activated replicons.

Further to replication-related chromatin loops, which are indirectly comprised by our model via the inhibition distance *d*_i_, the model also includes chromatin loops from the random loop model approximation of nuclear chromatin folding^52^. The size of chromatin loops originating from the random loop model (at least 2 Mbp)^52^ is much bigger than the size of the replication-related chromatin loops which corresponds to the view that chromatin loops are formed both as a result of polymer properties of chromatin fiber and involvement of DNA into nuclear processes^14^,^32^,^57^. Accordingly, similarly to other models of nuclear chromatin organization (Random walk/giant loop scales in the model by Sachs *et al*.^58^; multiloop subcompartments and giant loop domains in Munkel and Langowsky^32^) our model is based on different scales of chromatin looping, where the loops arise from both physical and functional properties of chromatin fibres^57^.

Another important ingredient of our model is the presence of a limiting factor that restricts the total number of replication forks active at any given time during S-phase. Other authors^22^,^59^ already established that a limiting factor is needed to obtain realistic origin activation profiles and synthesis rates in models of mammalian DNA replication. After an initial mono-exponential increase during the first hour, the limiting factor was kept at the constant value 12,000, which agrees with our count of 4,000–6,000 replicons^26^. We arrive at the same number of available limiting factors when calculating the total number of replication forks based on the duration of S-phase, the size of the genome and the fork speed obtained from our experimental characterization of HeLa cells^26^. This means that the limiting factor is fixed by two consistent experimental measurements. Using a constant limiting factor has the advantage that it is simpler than other approaches, which require a growing limiting factor^33^,^59^ or a time-dependent firing rate^60^,^61^ to control the replication rate.

Unlike previous models^14^,^59^,^60^, we explicitly used the specific chromatin layout on the scale of chromatin zones (euchromatin, facultative and constitutive heterochromatin) of human cells by modeling each HeLa chromosome like the corresponding human chromosome. We found that on the scale of chromatin zones not all details matter for the replication timing and the number of 1D replication clusters, as long as both firing types are enabled and the distribution of chromatin zone sizes has most of its weight between 1 and 6 Mbp (Supplementary Fig. 2). However, a more detailed probability map for initiation events as used by Gindin *et al*.^34^ enhances the correlation with experimental replication timing.

While on average euchromatic regions are replicated during early and heterochromatic regions during late S-phase, the exact time at which a specific site is replicated varies between individual simulations in our model, in agreement with the empirical observation that it varies in otherwise identical cells *in vivo*^19^,^62^. Cayrou *et al*.^19^ explained this observation with the ‘flexible replicon' model, which involves spontaneous firing and silencing of origins in the vicinity of firing events similarly to our model, but postulates preexisting clusters of origins, which our model does not require. Instead, clusters of replication forks are a result of the domino-like replication progression.

To relate the results of our 1D replication model to the characteristic foci patterns observed in fluorescence microscopy, we represented the fork positions derived from the replication model on a 3D chromatin conformation^51^,^52^. The simulated ‘in silico microscopy' images reproduce three major S-phase patterns observed in fluorescent microscopy^26^ (Fig. 4): the homogenous distribution of early RFi, the characteristic mid S-phase RFi at the nuclear and nucleolar periphery, where the facultative heterochromatin is located, and the clustered foci of late S-phase.

Higher compaction of facultative and constitutive heterochromatin were accounted for by introducing bigger values of spring constants into the model. More compact state of heterochromatin and accumulation of 1D replication fork clusters in heterochromatin was sufficient to reproduce characteristic complex RFi of late S-phase. Recent studies by high-resolution chromosome conformation capture confirm association between open and closed 3D chromatin structures with early and late replicating DNA^42^.

After stochastic activation of origins in euchromatic regions at the onset of S-phase, transition between early and late replication is observed within our model as the mid S-phase pattern, likely corresponding to replication timing of transition regions described by Pope *et al*.^42^.

The 3D RFi dynamics was generally reproduced using the same values for replication fork speed and distance parameters for induced firing and firing inhibition for the whole genome. The above parameters can potentially vary between individual genomic locations. When the corresponding data on chromatin organization and DNA replication dynamics is available for particular genomic segments, this information can be included into the model to better reproduce DNA replication dynamics in these parts of genome.

Similarly, parameters of our model can be adapted for a potential use in embryonic replication. There are several distinctive features of DNA replication in metazoan embryos including very fast (ca 20 min) S-phase, small replicon and chromatin loops sizes^14^. Therefore in case of embryonic replication the inhibition distance and limiting factor values should be changed and slower early S-phase should be excluded from the model.

To conclude, the experimentally observed spatio-temporal characteristics of DNA replication in somatic cells can be reproduced by a combination of 1D replication initiation/progression rules and random folding of DNA in the nucleus. Our model provides a minimal theoretical framework for a comprehensive description of S-phase dynamics in four-dimension including the complete genome duplication, overall S-phase duration, constant synthesis intensity, the timing profiles and the 3D patterns of individual replicons spatially similar to those observed experimentally^26^.

## Methods

All simulations underlying this publication were performed using the two simulation packages ‘replication' and ‘dna_metropolis', which were created by one of the authors. They are written in the C++11 standard of the C++ programming language and can be built and compiled using the GNU toolchain. Both packages, complete with source code (GPLv3 license) and installation instructions are available online at https://github.com/nleng/DNA-replication. Additionally, for illustrative purposes, the simulations can be performed online at http://sim.bio.tu-darmstadt.de together with an evaluation of the results.

### Simulation package ‘replication'

For the implementation of our replication model, we translated our algorithm into search, insertion and deletion operations on sorted lists. Unlike the algorithm proposed by Jun *et al*.^60^, who use two lists for the length of replicated and unreplicated regions, in our algorithm ordered lists are maintained for barriers, potential origins, left-going forks and right-going forks. The central data structure in the system is the event heap which is a binary heap data structure that at any given time contains all future collision events between the objects that are currently in the system (forks, chromosome barriers and chromatin zone transitions), sorted by time of occurrence. Thus the root element in the heap always holds the next event in the system. In each simulation step, the root element of the heap is removed and time is advanced to its time of occurrence.

If the removal triggers a chromatin zone boundary crossing or a firing event (because a limiting factor has been freed), then the addition and removal of future collision events becomes necessary. To keep such operations efficient, ordered lists are maintained for barriers, potential origins, left-going forks and right-going forks. These lists are implemented using a special red-black tree that, in addition to standard red-black tree behaviour, allows indexed element access scaling *O*(ln*N*) with the number of elements *N* (all nodes keep track of the number of their children).

For instance, if it is determined that an origin has to be fired, a random origin is picked from the available origins and checked if it has been passively replicated by the active forks. If not, its relative firing probability (a value between 0 and 1) is determined by the maximum of the spontaneous and induced firing probabilities and a random number between 0 and 1 is drawn. Should the random number be lower than the probability, the origin is fired, otherwise the process is repeated. If the origins lies inside the inhibition distance of an active fork, its firing probability is set to zero. Firing of the origin means that two forks, one in each direction, will be created, which have to be inserted into the fork lists, and for which collision events have to be calculated.

Experimental data suggest that the total number of replicons is between 4,000 and 6,000 (ref. 26). We consider a replicon to consist of two forks, meaning that the number of active replication forks is ∼12,000. Accordingly, in our model, the maximum value of the number of replication forks is set to *L*_max_=12,000. With this value, the total genome replication time obtained in the computer simulation agrees with the empirically found S-phase duration^26^. To model the increase of the limiting factor *L*(*t*) in the beginning of S-phase, we used the function


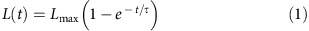


with *τ*=15 min, as obtained from the dynamics of RFi numbers measured in live HeLa Kyoto cells in the beginning of S-phase. The function as well as the experimental data is shown in Fig. 2a. A model with a linearly increasing number of limiting factors as proposed before^33^,^35^ would not fit the data as well as the exponential relaxation used in our model.

A fork moves along the DNA until it collides with another fork that ‘moves' in the opposite direction, whereupon both forks annihilate. As both the activation of an origin as well as annihilation require two forks, they do not only appear in pairs but are also removed in pairs, freeing two limiting factors. We assume that forks travel freely from one chromatin type into another, but are stopped at the boundaries between chromosomes, setting one limiting factor free.

### Package ‘dna_metropolis'

In the random loop model, a polymer (that is, the DNA) is approximated as a chain of beads with harmonic springs between adjacent beads without volume exclusion (Gaussian chain). Non-adjacent beads are linked randomly, such that loops are generated at an average incidence of 5 loops per 10 Mbp. Because this random linking generates loops on all size scales (that is, possibly connecting any two positions on a chromosome), they serve to restrict chromosomes to the limited volume. Movement of beads is restricted to an oblate ellipsoid with two horizontal semi-axes of *r*_x_=7.5 μm and *r*_y_=5 μm and a vertical semiaxis of *r*_z_=3.5 μm, which models the volume of an average nucleus.

When we laid out the rationale for origin firing inhibition, we based our argument on looped domains on a *d*_i_=55 kbp, which equals the lower estimate for the domain scale^63^,^64^,^65^. Since the inter-bead distance used in our random loop model simulations is 100 kbp, these domains are not resolved in the Monte Carlo model results and should not be confused with the loops of the random loop model. These latter loops, which have an average size of 2 Mbp, participate in the higher order chromatin organization.

In a previous study of human DNA by Mateos-Langerak *et al*.^52^, different linking probabilities were used to model differences in displacement for transcriptionally active and inactive regions. However, in using such linking probability variations for euochromatin, facultative heterochromatin and constitutive heterochromatin, we noticed that beside from the uneven distribution on the scale of the whole cell, there was no discernible difference in the micro arrangement of the three chromatin types and thus no formation of distinct 3D RFi (Supplementary Fig. 3). We, therefore, used different spring constants for the three chromatin types and random linking instead to reflect the different degrees of compaction of the three types of chromatin.

HeLa karyotype data were used to generate the bead chains for all chromosomes. One necessary extension of the random loop model is the inclusion of the experimentally observed accumulation of facultative heterochromatin at the nuclear and nucleolar periphery. Thus the cell was simulated with two nucleoli, wherein the polymer chain is not allowed to enter, and the attraction of facultative heterochromatin was implemented as a pseudo gravitational potential. Additionally, a small repulsive force was introduced into the model to minimize the overlap of chromosome territories.

The potential for a Gaussian chain with *N*_beads_ beads with position **x**_*i*_ is:


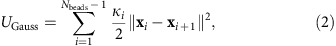


with the spring constant *κ*_*i*_ in our case being 1 × 10^−8^ for euchromatin, 5 × 10^−7^ for facultative and 3 × 10^−6^ for constitutive heterochromatin. The order of magnitude of these spring constants determines how compact the chromatin is structured. Therefore, the visibility of S-phase patterns in the in silico microscopy images, such as a homogenous distribution in early and RFi in late S-phase, is sensitive to changes in these parameters. *N*_beads_ varies between the chromosomes with a total number of 103,634 beads for the whole genome (one bead per 100 kbp). Connections between beads of different chromosomes are skipped. Random loop connections within chromosomes give an additional potential term:


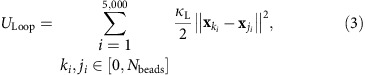


where the total number of 5,000 connections is based on a comparison of random loop model results with experimental genomic distance data by Mateos-Langerak *et al*.^52^. We chose an average loop size of 2 Mbp, which is towards the low end of their loop size estimate. The spring constant here is *κ*_L_=5 × 10^−7^.

In our model, cellular scaffolding and membrane interactions are implemented as two pseudo gravitational forces. First to ensure that each chromosome has its own nuclear territory, it is necessary to implement a small repulsive force (reversed gravity) between chromosomes. This effect was achieved in a previous model by defining local ‘effective temperatures' resulting from non-equilibrium activities such as gene transcription^66^. But as the chromosomal overlap is not a central aspect of our model, we pursue a simple approach with the following repulsive potential:


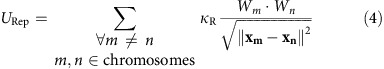


Here, vectors **x**_*m*_ and **x**_*n*_ are the centre positions of chromosomes *m* and *n*, *W*_*m*_ and *W*_*n*_ are the chromosome weights (that is, number of beads). In all simulations presented here, *κ*_R_=10^−4^ was used, which means that the per-bead contribution of the repulsive potential is significantly smaller than the contribution of the bead connection potential. Because a Gaussian chain without volume exclusion is used for each chromosome, the repulsive force is needed to avoid all chromosomes being distributed on top of each other.

Second, to generate the experimentally observed distribution of facultative heterochromatin at the nuclear and nucleolar periphery, a gravitational force between beads belonging to facultative heterochromatic zones and the nuclear membrane or the nucleolar membrane has been implemented. This additional potential is important for the visibility of a distinct mid S-phase pattern.


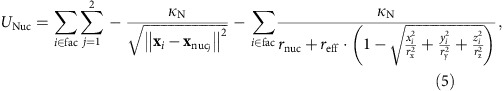


where **x**_*i*_=(*x*_*i*_, *y*_*i*_, *z*_*i*_) is the position of the *i*th bead, 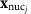 the position of the nuclei, 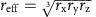 the effective ellipsoid radius and *r*_nuc_=1.0 μm has a value close to the nucleolar radii (1.2–1.5 μm) to prevent the potential from having infinite values and to generate a similar strength for both the nuclear and nucleolar periphery. Additionally, the same force was used for constitutive heterochromatin, but with reversed sign and lowered strength (*κ*_N_=30.0 for facultative and *κ*_N_=15.0 for constitutive heterochromatin).

For the total potential, the four terms are added together:


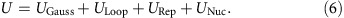


We use the standard Metropolis algorithm to let the beads relax into equilibrium with a temperature reservoir at 290 K. Replication fork positions from our replication model are then mapped onto the chromatin, thus generating a coordinate in 3D's for each fork.

### Cluster front speed

The slope of the replication timing curves is determined by the progression of induced firing and can be estimated by the following considerations. After the initial spontaneous firing event, a 1D replication cluster starts expanding. Once the limiting factor has reached its stationary value of *L*_max_=12,000, the average amount of DNA replicated within each cluster per unit time is given by *vL*_max_/*N*_c_, with *N*_c_ being the number of clusters. As long as the cluster consists of two fronts (early S-phase) ‘wave speed' of each front can be estimated as follows:


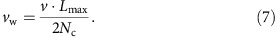


At the end of early S-phase (2.8 h), when the fork speed has reached its final value of *ν*=28 bp s^−1^, *v*_w_ has a value of ∼100 bp s^−1^, which matches the slopes of replication timing measurements reported in the literature^10^,^45^. It progressively increases as the number of 1D clusters declines.

### Image acquisition

HeLa Kyoto Cells (see Chagin *et al*.^26^) were grown on square coverslips to 60–80% confluence, washed and fixed with 3.7% freshly prepared formaldehyde solution. Immunofluorescence stainings were performed as described by Chagin *et al*.^26^. After rinsing with PBS the coverslips were stained with 100 ng ml^−1^ 4,6-diamidino-2-phenylindole (DAPI; Sigma). Samples were mounted in Vectashield (Vector Laboratories).

Single section 16-bit images of DAPI, green fluorescent protein/mCherry-PCNA fluorescence for several arbitrary fields were acquired using a Leica SP5 confocal microscope equipped with HCX PL APO lambda blue 40.0·1.25 OIL UV objective. Excitation of DAPI, green fluorescent protein or mCherry was performed with 405 nm (diode laser), 488 nm (Argon laser) or 543 nm (He-Ne laser) laser lines, respectively. The parameters of the system were adjusted to avoid saturation. Settings used were: 2,048 × 2,048 pixels (387.5 × 387.5 μm^2^) frame size, 8 airy unit pinhole diameter; 200 Hz scan speed.

3DSIM images (Fig. 4a and Supplementary Fig. 3a) were acquired and reconstructed as described in Chagin *et al*.^26^

### Image quantification

Integral DAPI intensities of individual nuclei in single images were quantified using the ImageJ ‘Analyze particle' command. The background signal was excluded by setting threshold at the level of intensity of the signal outside the nuclei.

The command generated a table containing integrated intensities of DAPI signal in the individual nuclei and returned the image of the outlines of the measured nuclei with the assigned numbers (Supplementary Fig. 1a).

That image was used as complementary data to the table with information on cell cycle stage of the cells: First, each of the nuclei was classified as early, middle or late S-phase or non-S-phase based on visual inspection of the proliferating cell nuclear antigen (PCNA) pattern (Supplementary Fig. 1b). The non-S-phase cells were separated into G1 and G2 subgroups based on stepwise increase in the DAPI signal (Supplementary Fig. 1c).

Average intensity of G1 and G2 nuclei was calculated and all measured values were normalized using:


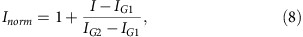


where *I* is integral intensity of an individual nucleus in an image, *I*_*G*1_ and *I*_*G*2_ are average intensities of G1 and G2 nuclei in the image, respectively, and *I*_norm_ is the normalized integral intensity of the nucleus. As a result of the normalization, the centres of the peaks for G1 and G2 nuclei were assigned to 1 and 2, respectively. This procedure was repeated for each field and the resulting normalized data were pooled and presented as a histogram with bin size 0.05. A total of 840 cells in five separate slide areas were analysed.

### Chromatin zone classification

An important feature of experimental DNA replication data is that early replication occurs preferentially in euchromatin (R-bands), whereas later replication occurs mostly in heterochromatin (G-bands). For this reason, a replication model must include the patterning of DNA into zones of different chromatin type^67^. In our model the DNA is conceived as a 1D string with a length of about 10^10^ base pairs, which is characteristic of the HeLa aneuploid genome^26^. Positions on the DNA are represented by a continuous variable.

Partitioning of the DNA into chromosomes is implemented by dividing the string into sections separated by barriers, which cannot be overcome by replication forks and block induced firing events. In contrast, replication forks can move through boundaries between eu- and heterochromatin zones. Therefore, the zones only differ with respect to their accessibility at the beginning of S-phase.

The sizes, positions and types of the chromatin zones were derived from human genome (hg19) Giemsa band data of the UCSC Genome Browser project (863 entries)^55^, because the staining values indicate the compaction of the local chromatin structure. Chromatin zones with zero Giemsa staining (gneg) were classified as euchromatin. Those with light staining (gpos25 or gpos50) as facultative heterochromatin. All other staining values (gpos75, gpos100, acen, gvar and stalk) were interpreted as constitutive heterochromatin. As an exception, the inactive X chromosome was simulated as 100% facultative heterochromatin to include experimental observations^68^. To adjust the model to HeLa cells, we added extra copies of those chromosomes that are contained more than twice in HeLa cells resulting in a total number of 76 chromosomes. The exact number for each chromosome is shown in Supplementary Table 2. Abnormal chromosomes were replaced by unaltered copies of their ancestral human chromosome. This resulted in 1,380 zones of euchromatin, 702 zones of facultative and 627 of constitutive heterochromatin. Due to differences in the average zone size the corresponding fractions of the total chromatin content are 42, 22 and 36%, respectively. The size distribution of the three chromatin zone types is shown in Supplementary Fig. 2.

### Data availability

The simulation source code (GPLv3 license) and installation instructions are available online at https://github.com/nleng/DNA-replication. Additionally the simulations can be performed online at http://sim.bio.tu-darmstadt.de.

## Additional information

**How to cite this article:** Löb, D. *et al*. 3D replicon distributions arise from stochastic initiation and domino-like DNA replication progression. *Nat. Commun.* 7:11207 doi: 10.1038/ncomms11207 (2016).

## Supplementary Material

Supplementary Information Supplementary Figures 1-6, Supplementary Tables 1-2, Supplementary Note 1 and Supplementary References

Supplementary Movie 1 2D projection of the Fork movement within the nucleus. The forks are colored depending on the chromatin type (blue: euchromatin, green: facultative heterochromatin, red: constitutive heterochromatin).

Supplementary Movie 2 Movie of an "in silico microscopy" maximum intensity z-projection throughout S-phase. A Gaussian blur was applied to imitate the limited experimental voxel sizes of 40x40x125 nm.

Supplementary Movie 3 Fork movement visualized in a rotating nucleus. The forks are colored depending on the chromatin type (blue: euchromatin, green: facultative heterochromatin, red: constitutive heterochromatin).

Supplementary Software Source code for 1D simulation of DNA replication and 3D simulation of chromatin folding.

## Figures and Tables

**Table 1:** Model parameters.

Parameter	Value	Underlying experimental data and consistency arguments
Genome size	*l*≈10 Gbp	Directly measured by Chagin *et al*.^26^
Number of eu-, facultative and constitutive heterochromatin zones	*N*_eu_=1,380*N*_fac_=702*N*_con_=627	Giemsa band data from the UCSC database (hg19)^55^
Number of potential origins	*N*_0_=500,000	Distances between MCM complexes^2^,^7^ and origins density in mouse cells^19^
Limiting factor	*L*_*max*_=12,000	Double the number of replicons^26^, consistency with fork speed and duration of S-phase
Initial limiting factor growth timescale	*τ*=15 min	Taken from replication foci number growth (Fig. 2a)
Maximum fork speed	*ν*=28 bp s^−1^	Directly measured by Chagin *et al*.^26^, consistency with limiting factor and duration of S-phase
Distance parameter of induced firing	*σ*=240 kbp	Distances between fired origins (Fig. 2b)
Distance parameter of firing inhibition	*d*_i_=55 kbp	Distances between fired origins (Fig. 2b), consistency with known size of looped domains^63^,^65^
Spontaneous firing probabilities	*p*_eu_=0.8*p*_fac_=0.05*p*_con_=0.0	Determined by the visibility of a distinct mid S-phase pattern produced by the simulations.

All the parameters of our computer model. For each parameter, the known/measured quantities from which its value is determined are listed. With the exception of *σ*, *d*_i_, *p*_eu_, *p*_fac_ and *p*_con_ the experimental values for all parameters were inserted into the model *a priori*.
