# Supplementary material for: 3D replicon distributions arise from stochastic initiation and domino-like DNA replication progression
Source: Nat Commun. 2016 Apr 7;7:11207. doi: 10.1038/ncomms11207 (PMC4829661; doi:10.1038/ncomms11207)
Supplement: Supplementary Information — Supplementary Figures 1-6, Supplementary Tables 1-2, Supplementary Note 1 and Supplementary References [file ncomms11207-s1.pdf]

Supplementary Figures

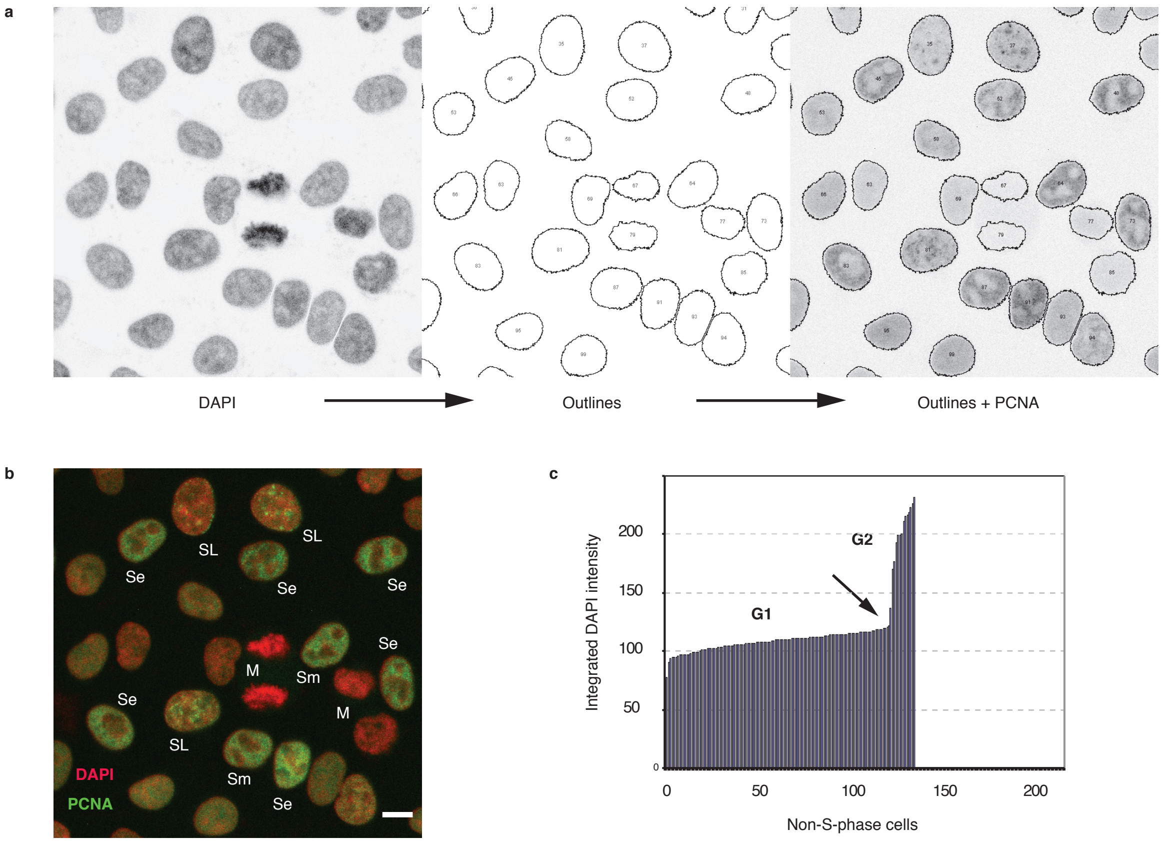

Supplementary Figure 1. Quantification of DNA content corresponding to the three major S-phase patterns. Nuclei of HeLa Kyoto mCherryPCNA cells were stained with DAPI. A total of 840 cells in 5 separate slide areas were analyzed. **(a)** Procedure for assigning DNA contents of individual cells with S-phase patterns. **(b)** Cells in early, middle and late S-phase were classified based on characteristic features of the PCNA distribution: uniform nucleoplasmic foci, perinucleoar foci rings and bright foci clusters, respectively. Note the relatively high DAPI intensity and absence of PCNA signal in mitotic nuclei. **(c)** Classification of non-S-phase cells into G1 and G2 populations based on the sharp increase in DAPI intensity. **(a)** and **(b)** show a small area of the field used for the analysis in **(c)**. The scale bar is $10\,\mu$m, this data was also used to estimate the nuclear sizes and shapes for the 3D model.

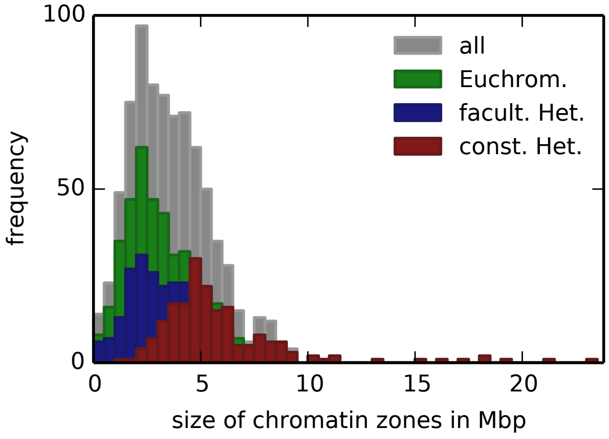

Supplementary Figure 2. Size distribution of chromatin zones. Human genome Giemsa and data of the UCSC Genome Browser project[1] was used to determine the sizes, positions and types of the chromatin zones. Zero staining was interpreted as euchromatin, light staining as facultative heterochromatin and dark staining as constitutive heterochromatin. The average chromatin zone sizes are 3.1 Mbp for euchromatin, 3.3 Mbp for facultative and 5.9 Mbp for constitutive heterochromatin respectively.

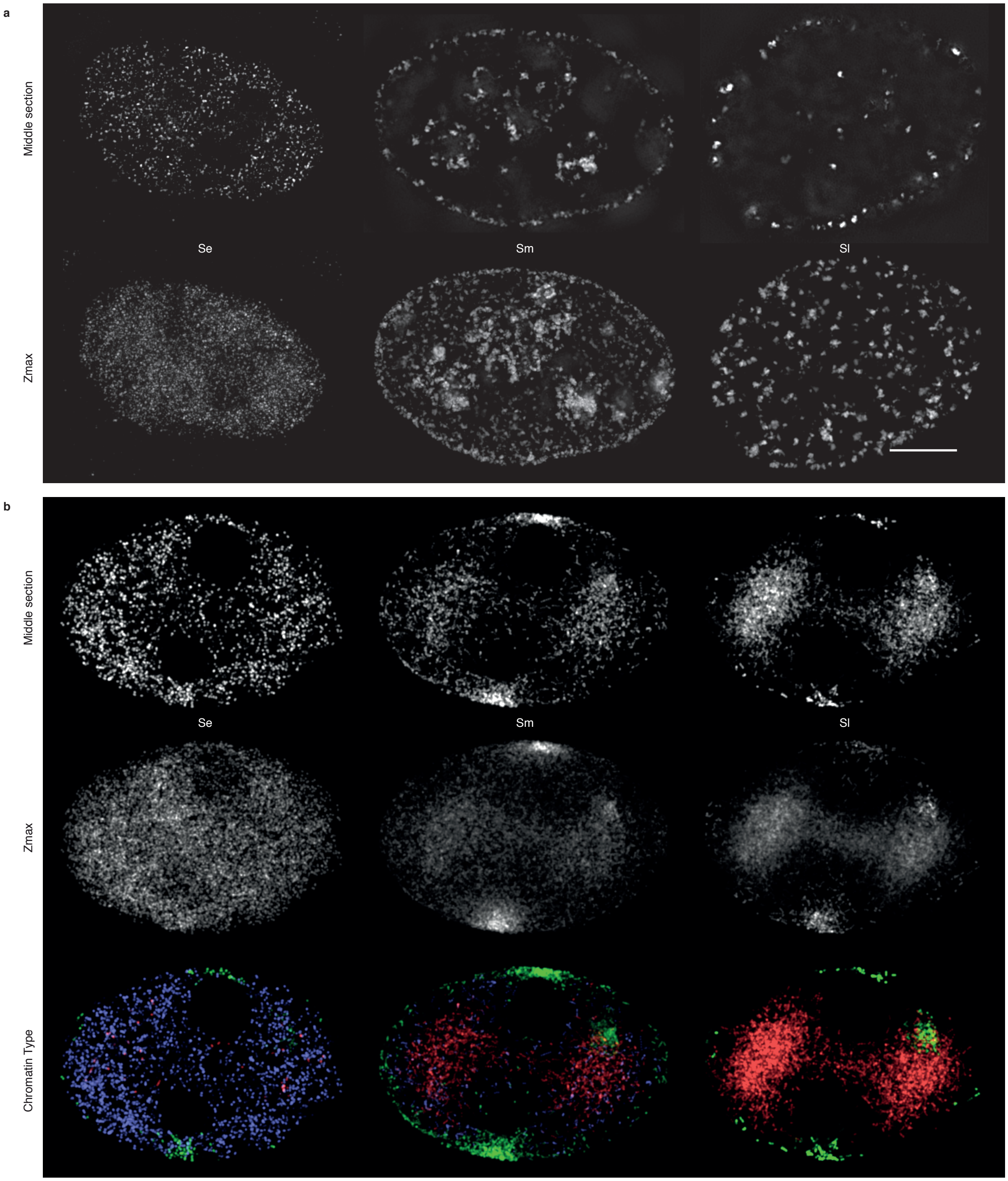

Supplementary Figure 3. Microscopy-like Images. 3D projection for the random loop model parameters used by Mateos-Langerak.[2] Both chromatin types have the same spring constant but the number of connections within them is different. Consistent with that publication, the total number of connections is 5,000, and the relative connection portions are 7/16 for constitutive Heterochromatin, 5/16 for facultative Heterochromatin, 3/16 for Euchromatin and 1/16 for inter-chromatin connections. Using these parameters, no clearly discernible formation of 3D foci is observed. **(a)** Experimental maximum intensity z-projections and middle section images of GFP-tagged PCNA inHeLa cells during early, middle and late S-phase (as described by Chagin et al.,[3] scale bar: 5 μm). **(b)** Corresponding "in silico microscopy" images. In the last row the simulated fork positions are marked depending on the chromatin type (blue: euchromatin, green: facultative heterochromatin, red: constitutive heterochromatin). Images for different parameters and chromatin distributions can be created online at http://sim.bio.tu-darmstadt.de. See also Supplementary Movies 1-3 for a visualization of the fork movement within the nucleus.

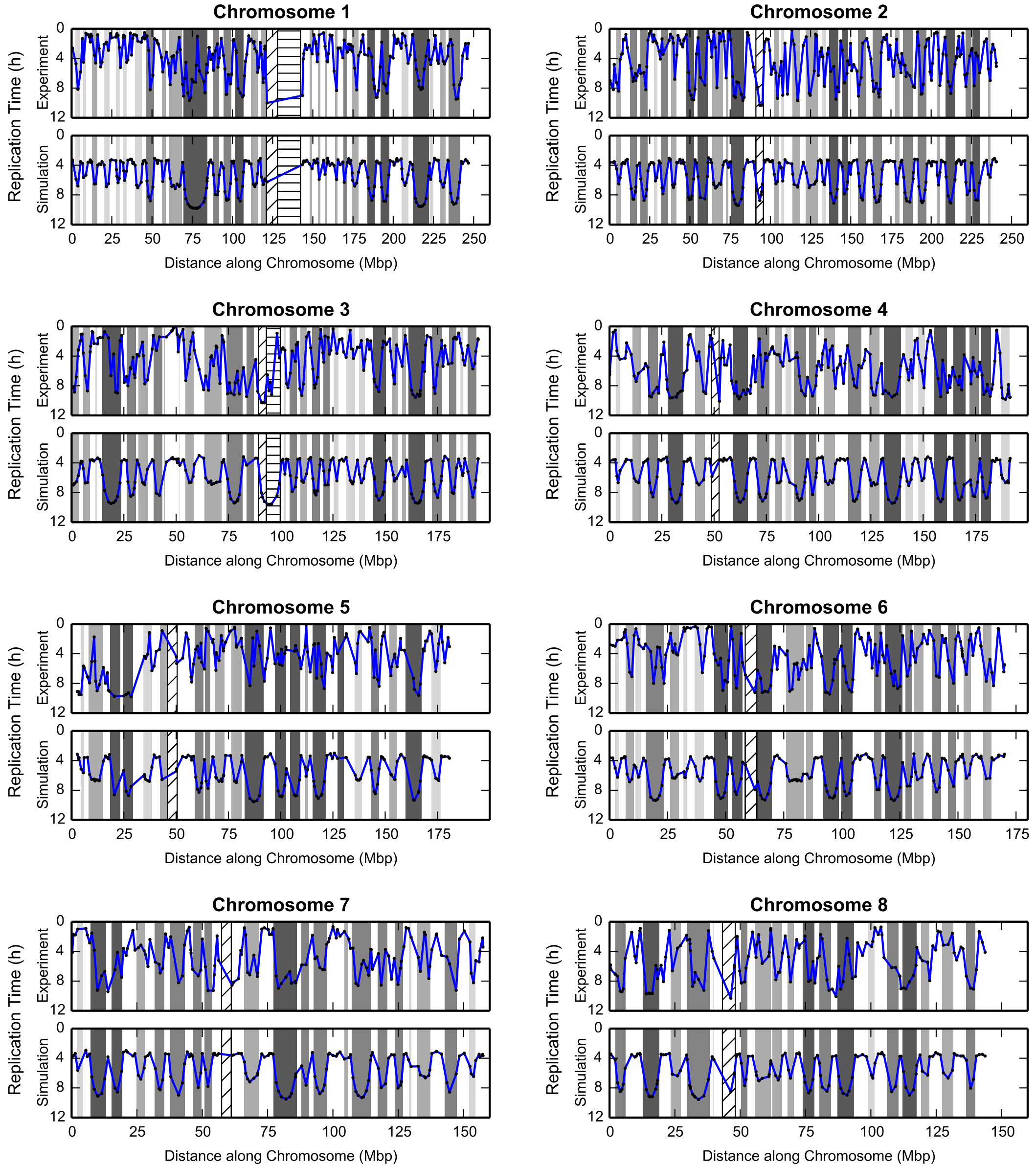

Supplementary Figure 4. Replication timing. Comparison of the replication timing of our model with data from the ENCODE project[4] (cell type GM12878) for chromosomes 1-8. Sampling positions are identical to the positions in the experimental data. For individual simulations, the euchromatic peaks start at time zero, but because of the specific sampling positions and averaging over 100 simulations, the displayed peaks are less extreme. The Background indicates the Giemsa staining, where white regions are interpreted as euchromatin and shaded regions as facultative or constitutive heterochromatin. The centromere is indicated as a striped pattern.

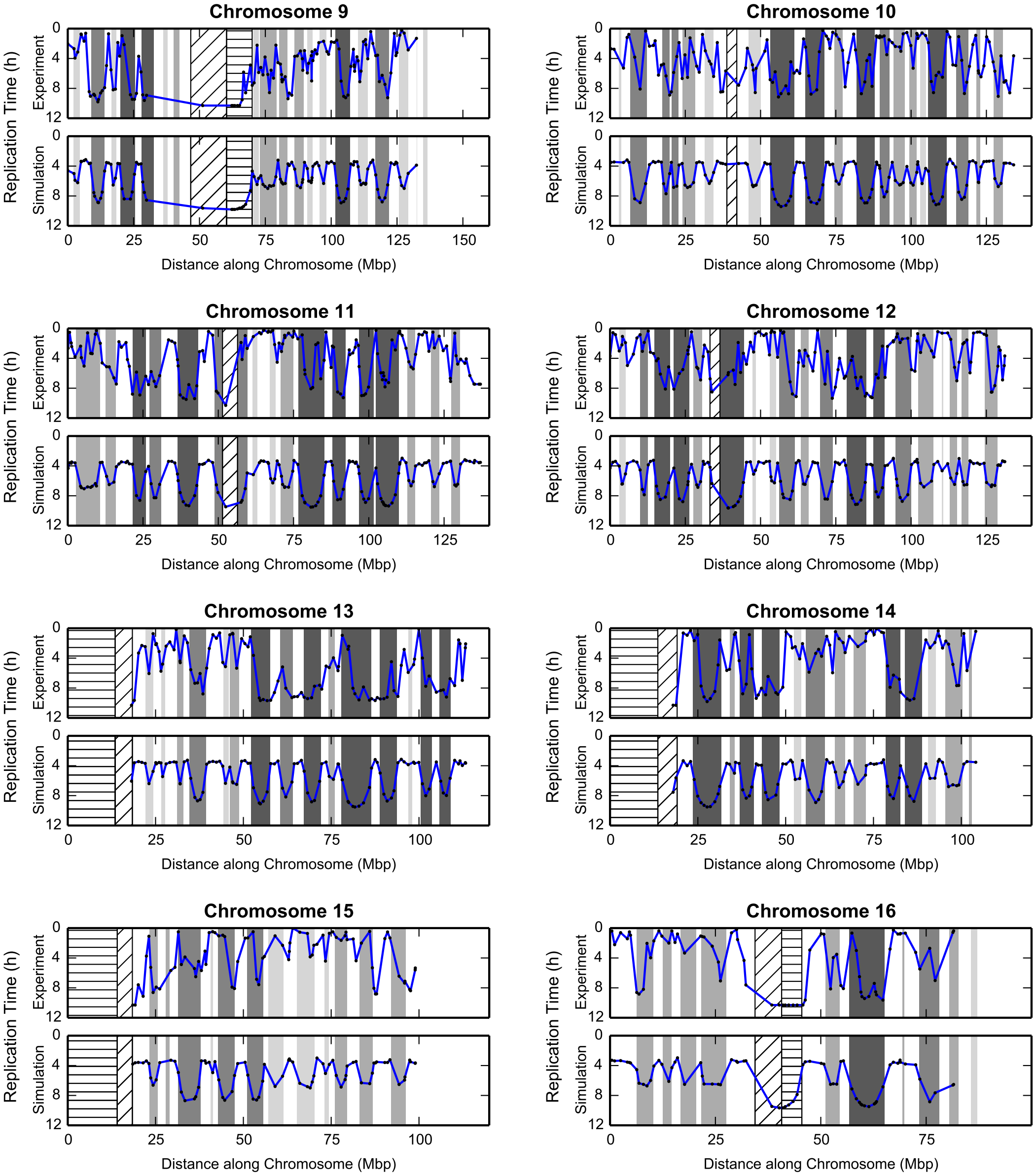

Supplementary Figure 5. Replication timing comparison for chromosomes 9-16.

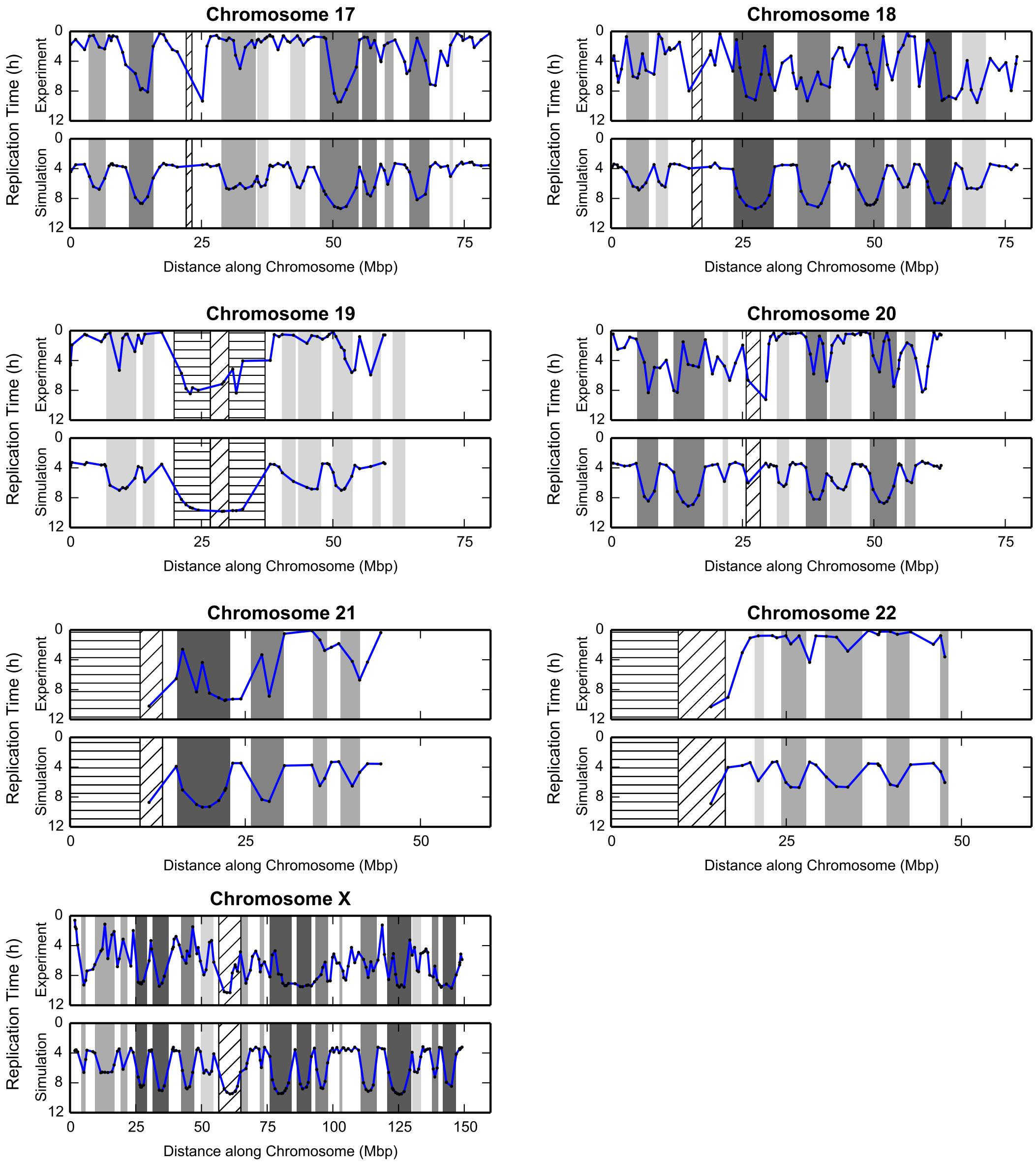

Supplementary Figure 6. Replication timing comparison for chromosomes 17-22 and the X chromosome.

Supplementary Tables

Supplementary Table 1. Average correlation coefficients for simulations and experimental data.

data type	Simulation	Woodfine	GM12878	Helas3	K562
**Simulation**	1.0	0.29	0.41	0.32	0.40
**Woodfine**		1.0	0.47	0.34	0.42
**GM12878**			1.0	0.74	0.83
**Helas3**				1.0	0.72
**K562**					1.0

Average over 23 chromosomes of the Pearson's correlation coefficients between the timing of sample positions in the model and in replication timing measurements from various sources (ENCODE project[4] and Woodfine et al.[5]). For all experiments the same chromosomal positions were used as by Woodfine et al.[5] (1 Mbp resolution).

Supplementary Table 2. Correlation coefficients for all chromosomes.

Chromosome	Duplicates	Correlation Coefficient
1	5	0.50
2	3	0.55
3	3	0.54
4	2	0.46
5	6	0.15
6	3	0.60
7	5	0.56
8	3	0.50
9	5	0.52
10	3	0.38
11	3	0.49
12	3	0.19
13	3	0.43
14	3	0.52
15	3	-0.05
16	3	0.63
17	4	0.43
18	2	0.21
19	3	0.65
20	3	0.16
21	3	0.44
22	3	0.27
23	2	0.41

Second column: Chromosomal duplicates used to model HeLa cells with a total number of 76 chromosomes.[6] Third column: Pearson's correlation coefficients between the timing of sample positions in the model and in measurements from the ENCODE project[4] for 23 human chromosomes with a resolution of 1 Mbp. The theoretical values used were averaged over 100 simulations.

Supplementary Note 1

We repeated the comparison of replication timing data from the ENCODE project[4] (cell type GM12878) and our model for 23 human chromosomes. The same level of agreement as for chromosome 6 was found for all chromosomes except chromosomes 9, 16 and the X chromosome. In chromosomes 9 and 16, the experimental data shows early replication of larger heterochromatic regions, whereas in the X chromosome data, the overall differences in replication timing for euchromatin and heterochromatin are much less pronounced. Possible reasons for this could be either epigenetic modifications regulating the replication of these chromosomes or the experiment statistics. The Pearson's correlation coefficients for all chromosomes and figures analogous to Figure 3e for all chromosomes are shown in Supplementary Table 2 and the comparisons are shown in Supplementary Figures 4-6.

We also compared the replication timing data of our simulation to data from three cell types measured in the ENCODE project[4] as well as data from Woodfine et al.[5] As shown in Supplementary Table 1 the correlation coefficients between experimental replication timing data (at the resolution of the Woodfine data[5]) varies between 0.34 and 0.83. The correlation between the averaged over 100 simulations and experimental data is lower (between 0.29 and 0.41). We ascribe this to the randomness of the simulated processes and the limited resolution of chromatin zones.

References

1. Dreszer, T. R. *et al.* The UCSC Genome Browser database: extensions and updates 2011. *Nucleic Acids Res* **40**, D918–D923 (2012).

2. Mateos-Langerak, J. *et al.* Spatially confined folding of chromatin in the interphase nucleus. *Proc Natl Acad Sci U S A* **106**, 3812–3817 (2009).

3. Chagin, V. O. *et al.* 4D Visualization of Replication Foci in Mammalian Cells Corresponding to Individual Replicons. *Manuscript co-submitted* .

4. Consortium, E. P. *et al.* An integrated encyclopedia of DNA elements in the human genome. *Nature* **489**, 57–74 (2012).

5. Woodfine, K. *et al.* Replication timing of the human genome. *Human Molecular Genetics* **13**, 191–202 (2004).

6. Macville, M. *et al.* Comprehensive and Definitive Molecular Cytogenetic Characterization of HeLa Cells by Spectral Karyotyping. *Cancer Research* **59**, 141–150 (1999).
